# A Review of Synthetic Image Data and Its Use in Computer Vision

**DOI:** 10.3390/jimaging8110310

**Published:** 2022-11-21

**Authors:** Keith Man, Javaan Chahl

**Affiliations:** UniSA STEM, University of South Australia, Mawson Lakes, SA 5095, Australia

**Keywords:** computer vision, image synthesis, synthetic image data, synthetic data generation

## Abstract

Development of computer vision algorithms using convolutional neural networks and deep learning has necessitated ever greater amounts of annotated and labelled data to produce high performance models. Large, public data sets have been instrumental in pushing forward computer vision by providing the data necessary for training. However, many computer vision applications cannot rely on general image data provided in the available public datasets to train models, instead requiring labelled image data that is not readily available in the public domain on a large scale. At the same time, acquiring such data from the real world can be difficult, costly to obtain, and manual labour intensive to label in large quantities. Because of this, synthetic image data has been pushed to the forefront as a potentially faster and cheaper alternative to collecting and annotating real data. This review provides general overview of types of synthetic image data, as categorised by synthesised output, common methods of synthesising different types of image data, existing applications and logical extensions, performance of synthetic image data in different applications and the associated difficulties in assessing data performance, and areas for further research.

## 1. Introduction

Modern approaches to computer vision primarily center around the use convolutional neural networks (CNN) and deep learning networks to train image processing models, methods which necessitate large amounts of labelled data and significant computational resources for training, while it is possible to use unlabelled data via unsupervised learning to train some computer vision models, the resulting performance is typically inferior to training via supervised learning and, in some applications, can fail to produce a model with meaningful performance [1]. The need for large quantities of labelled data makes it difficult for computer vision to be utilised in applications where the collection of large amounts of data is impractical, labelling data is costly, or a combination of both. Medical applications struggle with large scale data collection, crowd counting annotation remains a labour intensive task when done manually, and niche applications such as vital sign detection from drones suffers from both. Harder still is ensuring that the collected data is of sufficient quality and diversity to train a robust computer vision model to the performance level required by the application. These difficulties in image data acquisition has seen an increase in interest in synthetic image data as a potentially cheaper and more accessible alternative to acquiring real data for training, while multiple data types used in the field of computer vision, this review paper is primarily focused on evaluating the use of camera like image data and methods of generating such data synthetically. As such, the synthetic generation of data types that have seen use in computer vision, such as radar scans, sonar scans, and lidar point clouds are not are not considered.

Synthetic image data is defined in this review as any image data that is either artificially created by modifying real image data or captured from synthetic environments. This can take many forms, including the digital manipulation of real data [2] and the capture of images from synthetic virtual environments [3]. The visual look of synthetic image data also varies between fields of application, from composite imagery [4,5,6] to photo-realistic computer generated images [7,8,9,10,11,12].

The key advantage of synthesising image data, and the primary reason that makes the generation of data faster and cheaper, is that a properly set up image synthesis pipeline is capable of automating the data generation and labelling process at a comparatively low cost to manual labour. That said, it is important to note that not every method of image synthesis provides automatic data labelling and some methods still require significant amounts of human labour before the synthesis process can be automated. The use of the terms “faster” and “cheaper” to describe synthetic image data is mainly in comparison to the collection of real data for a given application. In many computer vision applications, obtaining large real datasets can be difficult and the manual labour required to label and annotate the raw data to the required level of quality comes at a significant cost. Large scale data collection can face limitations due privacy concerns, legal restrictions, and practical limitations, all of which reduce the amount of data that can be collected for training purposes. Manual annotation of data is also a time consuming task that becomes highly impractical for large datasets or datasets that require large numbers of labels per data instance, such as for crowd counting [13,14]. Even if manual labelling large data sets is possible, human error will inevitably cause some decrease in data quality as the data set size increases, resulting in a negative effect on training [15], while it is possible to train an algorithm to automatically label data in place of a human, training such an algorithm necessitates large quantities of labelled data for training to begin with, making it somewhat of a circular problem. Synthesising image data provides significantly more control over the resulting dataset, allowing for higher labelling accuracy, and object and environment information that would otherwise be difficult to collect in addition to image data in the real world.

Naturally, it is important to consider the limitations of synthetic image data as well. Data bias is a known issue with synthesised image data, often caused by inherent bias in the input parameters for data generation, while synthetic data may not necessarily have any direct relation to real objects, environments, or people, real data is often still needed to form a reference on which synthetic data is created and biases in the real data will influence the synthesised output. The domain gap between synthetic image data and real data is also an issue commonly noted in research, with models trained only on synthetic data typically displaying a significant drop in performance when tested on real data. Domain gap is not a problem unique to synthetic data as it is also present when training and testing on different sets of real data. However, the domain gap between synthetic and real data is more noticeable when testing the performance of models trained only on synthetic data. Finally, a less discussed limitation is the computational requirements to generate synthetic image data. In order to generate large synthetic data sets within reasonable time frames, significant computational power is required, computational power that may not be readily available to some people wanting to utilise computer vision in their field of work, while many papers have discussed the advantages and limitations of synthetic data in computer vision, much less has been written about the computational resources and time required to generate the data sets that were used. Nevertheless, this remains an important point of discussion as these two factors can greatly impact the practicality of using synthetic data in many applications.

Past reviews on synthetic data for computer vision have evaluated the use of synthetic data focusing on the image generation process [16] or the use of synthetic image data for specific applications such as navigating urban traffic environments [17], pedestrian detection [18], text-to-image synthesis [19]. The goal of this review paper is to categorise existing types of synthetic image data by output, review methods used to synthesise such data, discuss the effectiveness of synthetic data in various computer vision tasks, logical extensions to current use of synthetic data, and identify research gaps that may lead to future research. Section 2 covers the difficulties and associated costs of obtaining real data for computer vision applications and the reasons why synthetic image data has grown in popularity as an alternative source of data. Section 3 covers the different types of synthetic data used in computer vision as categorised by method of synthesis. Section 4 discusses the methodologies used to synthesise image data as well as the strong points and weaknesses of each methodology. Section 5 examines the current effectiveness of synthetic data in various computer vision tasks, comparing types of synthetic data where applicable. Lastly, Section 7 considers the research gaps and challenges in current image data synthesis methodologies and applications.

## 2. Computer Vision and Synthetic Image Data

The rise of CNN and deep learning networks in computer vision has necessitated ever larger amounts of image data for training and testing. Such image data is commonly stored in the form of photos and videos. The traditional method of obtaining image data for training, testing, and validation of neural networks has been to capture data from the real world, followed by manual annotation and labelling of the collected data. This methodology is relatively simple and cost effective for smaller image data sets, where there are a minimal number of key objects per image, the exact position of objects is not important, and only the general classification is needed. However, this methodology becomes increasingly more costly when scaled up to large data sets or datasets that require more detailed annotation.

The first problem is the collection of large amounts of data. It is possible to automate the collection of real world image data to a certain extent in applications that utilise fixed cameras or vehicle mounted cameras, but this is not the case in all computer vision applications. Collection of large datasets for applications such as facial recognition and medical scans can be very difficult for a range of reasons including privacy, cost, and other legal restrictions. It can also be difficult to reliably collect data of specific environmental conditions such as foggy roads, where the presence of fog is not something that can be typically created in a public space just for the sake of data collection.

The difficulty only increases when detailed data annotation is needed. Manual data annotation can be a slow and laborious task depending on what needs to be annotated and to what degree of accuracy and precision. Some data sets, such as ImageNet [20], are relatively simple to annotate manually as the images primarily need to be placed into categories based on what the primary object in focus is. However, the annotation of over a million images is a massively time consuming task, even using a large group of people. When considering the annotation of more complex details such as the number of people in a crowd, object poses, or the depth of objects, the cost effectiveness decreases significantly. Time and money are not the only concerns either, manual annotation quality tends to decrease when dealing with large data sets due to human error. In some applications, such as the previously mentioned crowd counting, it may not even be possible for a human to reliably count the number of people depending on image quality and crowd density.

For such applications, synthetic data provides two key benefits. The first benefit is that data generation can be automated. Generation of synthetic human faces with varying levels of realism has been possible for many years and has enabled the creation of data sets for facial recognition without the privacy concerns that come with taking photos of people or the time and cost required for many people to have their photo taken. The second benefit is that as long as the data synthesis model is able to keep track of various objects and features during the synthesis process, detailed automatic annotation is possible. Of course, it is important to note that although automatic annotation is possible, it is still dependant on how the data synthesis is set up. Detailed information on all objects within a virtual 3D environment is usually available in 3D modelling software or game engines, but if that information is not extracted as part of the data synthesis process then there is fundamentally no difference to collecting data from the real world.

These are the primary difficulties behind data collection and annotation for computer vision applications and the major benefits synthetic data can provide.

## 3. Types of Synthetic Imagery for Computer Vision

Synthetic image data can be broadly categorised into two types, synthetic composites and virtual synthetic data.

### 3.1. Synthetic Composite Imagery

Synthetic composite imagery refers to real image data that has been digitally manipulated or augmented to introduce elements that were not originally in the image data. This includes the digital manipulation of the image environment, introduction of synthetic objects into the image or the splicing of different real images into a new image.

**Figure 1:**
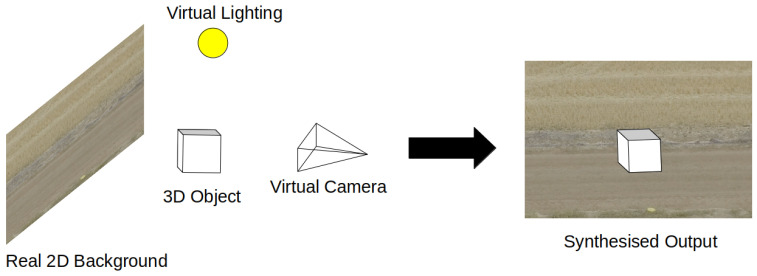
Synthesis via projection of synthetic 3D object onto real 2D background.

Synthetic composite datasets such as SURREAL [21] are created by projecting 3D synthetic objects or people into real background environments, Figure 1. This data type is often used in situations where the background environment contains enough useful or significant features that it is not worth the loss in domain shift or the effort to recreate the environment synthetically. The SURREAL dataset was primarily created to train networks on human depth estimation and part segmentation. As a result, the synthetic humans do not take into account the background environments they are placed in. The resulting scenes can be easily identified as synthesised by the human eye, but features the network needs to learn are attached to the human object, so the background simply serves as a way to reduce the domain gap to real data by increasing environmental diversity.

**Figure 2:**
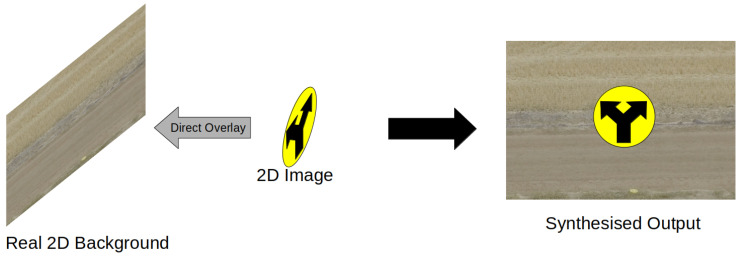
Synthesis via overlaying 2D image onto real 2D background.

Similarly the RarePlanes dataset [22] provides synthetic composite satellite imagery of aircraft at different airport locations. However, instead of projecting 3D objects onto a background, 2D images are directly overlaid onto the backgrounds, Figure 2. Satellite imagery is one of many fields of computer vision where it is difficult to obtain large data sets due to the nature of the image data required. The authors of the paper notes that there are no expansive permissively licensed synthetic data sets for such data. The RarePlanes dataset consists of a mix of real and synthetic satellite imagery that has had aerial images of planes overlaid on top, while Figure 2 notes the use of real 2D backgrounds, in practice, this can be extended to synthetic 2D backgrounds as well as it does not affect the overall process of overlaying 2D images onto a background. The synthetic data was created using the AI.Reverie platform, which used Unreal engine to create realistic synthetic data based off real world airports.

Large crowd data sets are resource intensive to annotate, both images and videos, with large numbers of people, in excess of 1000 people in some cases. People in crowds are also often not fully in view, potentially only having part of their head visible with the rest of their body obscured by the surroundings. Manual annotation can result in cases where data is not fully labelled due to the difficulty in doing so, thereby introducing data set bias. There are two common methods of synthesising crowd data. The first is to use 3D human models and either project them onto a 2D background or place them into a 3D virtual environment. In practice rendering scenes with over 1000 models would be highly computationally demanding, but if video data is needed, this is still the easiest method of generating crowd data. The second method is to use 2D overlays to project images of humans onto a 2D background. A paper on large crowd analysis using synthetic data [23] projected synthetic humans onto real scenes. The synthesis enabled illumination, movement and density of people to be controlled while providing ground truth information.

Data sets such as foggy scenes [2] use real data as a basis and digitally manipulate the image data in order to produce synthetic variations. Such data is created for applications where it is difficult to obtain data due to specific environmental requirements, but real environments and objects still hold enough value that it is not worth the effort of recreating the entire scene virtually to create the necessary data. In practice, this method of image synthesis can be considered an extension of overlaying 2D images onto a background, but instead of overlaying an image, a filter is used to project the required environmental conditions. Compared to 2D images, filters are also comparatively simpler to extend to video data if so required.

While all synthetic composites are image composites by definition, there are also some synthetic composites that do not use any synthetic objects or images in its creation. Image compositing works the same way as 2D image overlays, but uses labelled 2D objects from objects from one set of data and placing the object into scenes from other sets of data. This method of data synthesis tends to create data set with lower domain gap than virtual synthetic data sets, possibly due to domain randomisation increasing data diversity and improving generalisation [4].

The fish identification data set [24] is an example which uses instances of real fish cropped out from data collected using the Deep Vision system [25] and places them onto backgrounds from Deep Vision footage where no other fish or objects are present, in random orientations, positions, and sizes. The resultant composite image comprises of only real data, but is still considered synthetic data as the exact scene was not capture in the real world. The reason for the generation of such data is primarily the difficulty in annotating existing Deep Vision data. Generating synthetic data with known fish species allows for much cheaper labelled data and extracting fish from scenes where the species can be readily identified by a human is also a significantly less time consuming task than manually labelling the original Deep Vision data set.

Image synthesis could be considered an extreme version of image compositing where instead of extracting labelled objects and placing them into other scenes, image synthesis takes labelled object features and combines them with other labelled object features to produce a new object. Visually, the new object may look nothing like the objects from which the features were extracted, but from the perspective of a neural network, the synthesised object still contains all the necessary features to identify what the object is [6].

**Figure 3:**
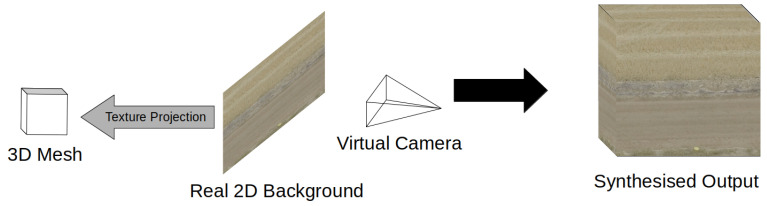
Synthesis via projection of real 2D image onto 3D mesh.

The KITTI-360 dataset [26] was created with the goal of augmenting the KITTI dataset [27] with more objects, increasing data efficiency for training. The paper noted that while 3D rendered virtual worlds were becoming more popular for producing urban environment data, the creation of such an environment requires significant human input before data can begin automatic generation. Instead, the paper proposed a process to integrate synthetic objects into real environments in a photo-realistic manner. By creating 360 degree environment maps, KITTI-360 was able to place high quality vehicle models into existing KITTI scenes with realistic lighting conditions. The models themselves are created by projecting 2D texture images onto 3D meshes, Figure 3, which are then projected onto backgrounds to give a realistic view of the object as the perspective on the object changes over the course of the video.

The SafeUAV synthetic dataset is rarer extension of mesh projection by projecting real 2D backgrounds to create a full 3D background [28]. SafeUAV uses a 3D mesh reconstruction of an urban environment in CityEngine before overlaying real photo data over the mesh, Figure 3. The result ends up warping the photo data significantly from ground angles but provides a reasonably similar view from above, which is all that is required as this dataset was generated for semantic segmentation and depth perception tasks from a drone.

**Figure 4:**
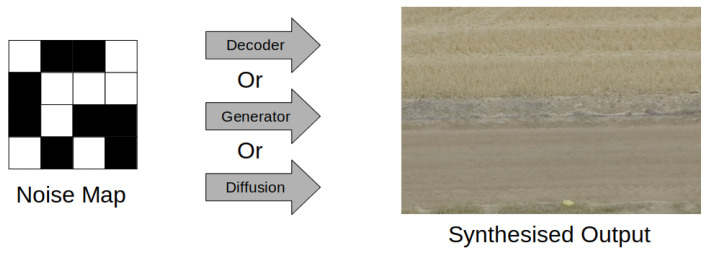
Synthesis via processing of noise maps.

The last type of synthetic composite imagery is less so an image composite and more of an extreme extension to digital manipulation. Images synthesised using variational autoencoders, generative adversarial networks, and diffusion models use noise maps as inputs to generate an image, Figure 4. By learning compressed representations of images, noise maps can be used by these models to extrapolate features into a complete image.

### 3.2. Virtual Synthetic Data

Virtual synthetic data refers to image data that is completely synthesised, containing no real data directly. This can apply to a wide range of synthetic image data from synthetic objects with patterned textures placed in front of artificial backgrounds to photorealistc 3D environments designed to emulate the real world. Based on the types of virtual synthetic data generation methodologies, virtual synthetic data can be categorised into three groups, virtual scenes, virtual environments, and virtual worlds. This categorisation is independent of the photo-realism of the data that is produced.

Virtual scenes are the simplest form of virtual synthetic data. Virtual scenes typically use the minimum amount of 2D and 3D objects to create a scene to capture synthetic image data. The generation of synthetic faces for facial recognition tasks using 3D morphable models or parametric models are an example of virtual scenes. Synthetic faces often do not generate anything below the neck, some models only generate a mask of the face. When viewed from the front, the faces can be captured and used as synthetic image data. If a background is required, it only needs to look correct from the viewing angle, in some situations a realistic background might not even be required. Observing the face from different angles or positions makes it clearly visible that the head is not a complete object. It is not a complete virtual environment, and for the purposes of such applications, a complete virtual environment is not required.

Virtual environments are a step above virtual scenes and comprise a complete 3D virtual construction of a specific environment. The environment could be the inside of a house or a pedestrian crossing. Either way, its goal is to enable the capture of image data from multiple perspectives without risking the degradation of data quality due to problems such as object artefacts. When viewed from outside, the virtual environment may still look incomplete, but within the environment, it is self consistent.

Virtual worlds are effectively virtual environments on a larger scale. Scenes outside a virtual environment that may have been flat 2D background are fully constructed with events occurring beyond the view of the virtual camera. This is most commonly found in virtual data captured from games that have pre-built large scale environments, such as from the game Grand Theft Auto V. Creating virtual worlds to collect such data is labour intensive, which is why collecting data from games with pre-built worlds is a common alternative. Virtual KITTI [29] is an example of a virtual world where the environment from parts of the KITTI dataset [27] was recreated digitally to produce a virtual copy of the KITTI dataset.

In the field of object detection, some research has moved towards highly photorealistic object renders to reduce the domain gap to the target domain. Other research has found that photorealism might not be the only method of reducing domain gap, instead by using domain randomization, where the objects of interest are placed into random non-realistic environments, it is possible to force a model to learn object features [30]. Compared to photorealistic objects, this type of synthetic data may not fit the target domain as well, but its generalisation means that it stands to have better average performance across multiple domains. Virtual synthetic data offers a way to create both photorealistic and photo-unrealistic environments that can be manipulated as required to produce the necessary image data.

Facial recognition has made significant progress over the past few years thanks to developments in deep learning networks and large scale data sets. However, it has started to become increasingly difficult to obtain larger data sets from the internet by trawling for faces due to labelling noise and privacy concerns. As a result, synthetic data has become the alternative to obtaining large data sets. The performance of networks trained on synthetic data for facial recognition has historically not been good, the domain gap has often been very large, resulting in poor real world performance. However, synthetic synthesised faces still offer the great benefit of avoiding issues with privacy and developments over the years have shown increased performance in face generation technology [31].

Moving past the generation of virtual synthetic data for standalone objects and faces, there are some applications that necessitate the construction of a larger virtual scene. In the scenario where a task such as pedestrian detection is required, but there is no existing real data to conduct network training or even domain adaptation, synthetic data is the only available method of sourcing any data to train a pedestrian detection model [3]. The problem is that synthetic data suffers from domain gaps with real data and without any real data, traditional methods of reducing the domain gap, such as mixing data or fine tuning after pre-training on synthetic data are not possible. In cases like this, the best option is to provide as much ground truth as possible from the virtual scene that has been constructed.

Vehicle re-identification is another field that can utilise virtual synthetic data in the scope of a virtual scene, while vehicle detection and identification is closely related to tasks such as urban driving, unlike urban driving, vehicle re-identification is primarily concerned with stationary vehicles and so synthetic vehicles can be placed into small virtual scenes for data collection. Similarities between vehicle types when viewed from different angles as well as the lack of differences between some vehicles types can cause many difficulties with real data. To address this issue, highly diverse data sets are required to learn specific features. However, even if such data is available, manually annotating such data is prohibitively expensive. Synthetic data provides an alternative source of large automatically labelled data that can also be generated from many different perspectives, allowing for much more diverse data sets than what might be normally available from the real world [32].

In cases where synthetic scenes are not sufficient to produce the data required for the application, synthetic worlds offer a much larger environment from which to capture data at a computational cost. Most virtual worlds are not fully utilised all the time. Instead virtual worlds allow for the capture of data in different environments, which can be useful in applications such as autonomous vehicles, while photo-realistic environments are not possible without the use of dedicated designers and significant rendering time, it is possible to generate more basic environments using city layout generation algorithms combined with pre-textured buildings, allowing for the creation of grid like city environments. The effect of photorealism on performance is substantial, but the biggest advantage of virtual synthesised environments lies in the automatic labeling of objects as well as complete control over environment variables [33].

While virtual synthetic data does not directly contain any real data, this does not mean that it cannot reference or replicate real data. The Virtual KITTI data set [29] is a fully synthetic recreation of a subset of the KITTI data set. The goal of creating a virtual copy of the KITTI data set was to provide evidence that models trained on real data would perform similarly in virtual environments and that pre-training on synthetic data should provide improvements in performance after fine tuning.

## 4. Methods of Data Synthesis

There are many means to generate synthetic data with different methodologies suited to different tasks and applications. However, most methods to generate synthetic data for computer vision can be grouped into the following categories.

### 4.1. Manual Generation

**Figure 5:**
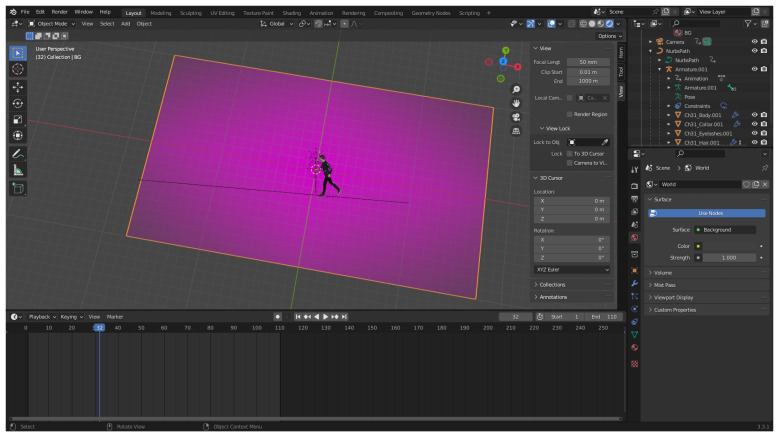
Manual creation of image and video data via 3D modelling software such as Blender.

Manual generation is the most basic form of data synthesis, Figure 5. Synthetic data, whether a composite image or 3D environment, is manually produced, instance by instance, to create a full data set. This is by far the most time consuming method of synthetic data generation and inevitably limits the quantity of data that can be created. Labelling and annotation of manually synthesised data will usually require more manual work, discarding the key benefits of synthesising data, which were supposed to be large scale data generation and automatic feature annotation. However, manual synthetic data generation does see use for specific situations.

One example is the reconstruction of 3D models based on 2D images [34]. The process of extracting information from objects in 2D images, such as pose and size, can be difficult without a ground truth to compare them with, while not exactly the same, 3D model reconstructions of objects in an image allows for a relatively accurate ground truth to be used in training. However, this does require rough manual recreations of objects from 2D images, as the problem is how to extract object information in the first place and training an algorithm to create 3D models of the object would require the problem to be already solved. If the number of 3D models required for training is not high, it may be more efficient to manually create the data as opposed to investing time and resources to automate the process. Another example is the creation of small 3D virtual environments for tasks such as localisation [35] and urban driving [26], while full 3D worlds can be automatically generated, the time and resources required to render a fully 3D world are too high to reach anything approaching the same level of detail as the real world within a practical time frame. In addition, there often is not a need to synthesise a complete 3D world, tasks like object detection are often focused on specific subsets of objects that exist within the world. Because of this, manually creating small virtual worlds with a specific design may be more beneficial than generating a large virtual world that might not be fully utilised. For the case of 3D worlds and environments, recent developments have seen a shift towards using game engines to build virtual worlds due to the tools and utilities that come with such software. In some cases, data can even be pulled from 3D environments that exist in games, forgoing the manual creation of a virtual environment entirely.

### 4.2. Generative Adversarial Networks, Variational Autoencoders, and Hybrid Networks

Moving from manual data synthesis to automated data synthesis, generative adversarial networks (GANs) are currently one of the most popular methods of generating 2D synthetic images. Applications range from generating data for computer vision tasks to the creation of AI art. This section covers the pre-cursor to GANs, variational autoencoders (VAEs), various well known GAN models, and some hybrid networks that utilise a combination of GANs and VAEs in some form.

**Figure 6:**
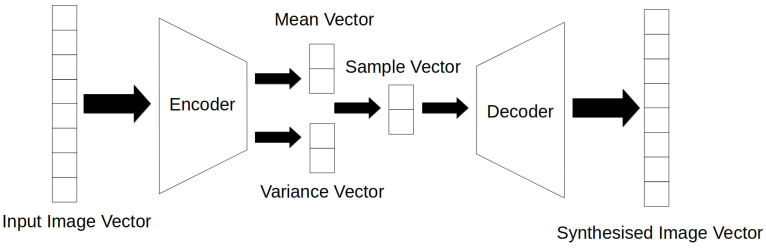
Variational Autoencoder training process.

VAEs [36] are a type of machine learning network that utilises two sub networks and served as a successor to classical autoencoders and a precursor to GANs in the field of image generation. Classical autoencoders consist of a encoder network and decoder network which function by taking an image, mapping it to a latent vector space and then reconstructing the original image. Training the autoencoder by making the input image and output target the same, an autoencoder learns how to generate higher resolution images from compressed representations. However, the performance of classical autoencoders proved to be insufficient for practical image generation and were eventually succeeded by VAEs. The main addition in VAE networks was the use of statistical distribution in the image encoding process. Classical autoencoders compressed inputs into a fixed sparse representation. On the other hand, VAEs encode images into a probability distribution across the latent space, from which the decoder samples random points to generate an output, Figure 6. This probability distribution encoding resulted in VAE networks being significantly more robust compared to classical autoencoders as all latent space samples could be decoded to a valid output. However, while VAE networks have found applications in tasks such as image denoising [37], feature extraction [38], and image compression [39], it sees limited use in image generation on its own. This is primarily due to its blurry output, particularly at higher resolutions, caused by its probability distribution encoding tending to ignore features that only consist of a few pixels. Instead, research into the use of VAEs for image generation has transitioned to hybrid models that utilise both VAEs and GANs.

**Figure 7:**
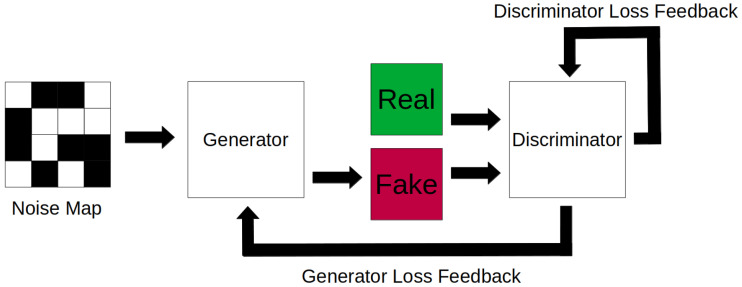
Basic model of a classical GAN training process.

First proposed in 2014 [40], the original GAN model also utilised two two sub networks, pitting a generator network against a discriminator network, Figure 7. The goal of a GAN is for the generator to produce synthetic data that the discriminator cannot tell apart from real data while the discriminator learns to distinguish generated data, hence adversarial. The generator learns by synthesising images from noise maps, learning better mappings to produce more realistic samples as the discriminator provides feedback. Vice versa, the discriminator learns from feedback for correct and incorrect classification of real and synthesised images. GANs can be further classified into conditional GANs [41], where the GAN is fed additional information such as class labels, and unconditional GANs, where the discriminator attempts to distinguish real and fake images independent of what the image actually is. In an ideal scenario, a generator will reach a state where its data is indistinguishable from real data. However, in practice, this rarely occurs and the generator and discriminator will typically maintain some form of balance, with convergence on the generator or discriminator indicating some form of failure. Compared to VAEs, which can be considered to use semi-supervised learning to generate images, as the target is the same as the input, GANs use unsupervised learning to generate its data.

As of 2018, there were over 450 named variants of GAN networks [42], too many to cover fully in a general review of synthetic data generation and usage. However, the more well known GAN models used to generate image data, BigGAN, CycleGAN, DALL-E 2 (and similar models), DCGAN, and StyleGAN will be covered with applications and differences in methodologies noted.

BigGAN and BigGAN-deep are a models based on SAGAN [43], a GAN design that added attention maps that allowed the generator and discriminator models to focus on features in different parts of an image. BigGAN utilises attention maps in addition to greatly scaling both parameters and batch size to increase the quality of generated images [44]. BigGAN is a conditional GAN as its generator and discriminator are both conditioned with class labels at time of training. When BigGAN was proposed, state of the art GAN models were showing weak Inception Scores [45] compared to real data, with the goal of BigGAN being to reduce the gap in image quality and resolution between GAN generated images and images from the ImageNet dataset. BigGAN demonstrated substantial improvements to Inception Score when trained and tested against ImageNet and JFT-300M datasets, showing good Inception Scores even at higher image resolutions of 256 × 256 and 512 × 512 pixels, which GANs traditionally have been weaker at. However, the authors noted important trade-offs to achieve these results. GANs are already inherently unstable and scaling GAN to BigGAN resulted in instabilities unique to GAN models of that scale, resulting in eventual training collapse and necessitating early stopping of training, while a combination of techniques was shown to be able to reduce those instabilities, complete training stability came at the cost of a significant cost to performance trade-off. Even without complete training stability, BigGAN still requires significant computational resources in comparison to smaller GAN models due to the overall increase in scale. Regardless of these trade-offs, BigGAN still remains the primary large scale GAN model and has since seen several derivatives with the goal of improving different aspects of the original model. LOGAN [46] introduced latent optimisation to BigGAN, improving performance on 128 × 128 pixel images compared to base BigGAN, although higher resolution performance was untested due to limited computational resources. Variants looking to reduce computation requirements, such as not-so-BigGAN [47] have also been developed as a significantly smaller compute budget alternative while attempting to reach competitive levels of performance.

CycleGAN is a unique variant of GAN model where the goal is not to generate new and unique image data, but to change the domain of an existing image to a new domain [48]. Image-to-image translation is a process in which an image is transformed to a different domain. Training of a computer vision model to achieve this task typically requires paired training data, where the same scene is depicted in both the input domain and target domain. As with many other computer vision tasks, obtaining paired image data for image-to-image translation is often a very complex, costly, and laborious task. CycleGAN was developed as a means to create image pairs for a set of unpaired data, allowing the creation of image pairs for domains such as painting styles, environmental conditions, and even conversions between animals. The name CycleGAN comes from the property of cycle consistency in the GAN model, which is to say that if an image is converted from one domain to another, converting the image back to the original domain should reproduce the initial image. This cycle consistency was achieved by utilising two generator/discriminator pairs for a total of four players in the GAN model. CycleGAN is trained by passing the domain converted output from the first generator through a second generator with the goal of reproducing the original image. By introducing cycle consistency loss, CycleGAN was able to reduce the space of mapping functions, preventing inputs from being converted to random permutations in the target domain. Interestingly, as the goal of CycleGAN is to convert an image’s domain and not to generate a completely new image from a noise pattern, it performs reasonably well on higher resolution images, such as photos from the Cityscapes dataset [49]. In comparison to other image-to-image translation models, the authors noted that there was a gap in performance compared to models trained on paired datasets, such as pix2pix [50], a gap that may require the use of semi-supervision in some form to close. However, CycleGAN’s methodology is still important as a model that pushed the boundaries of what could be done with unsupervised learning. CycleGAN has seen some derivative models, with most adapting it for use in specific applications, such as CycleGAN-VC [51,52,53] for voice conversion, res-cycle GAN [54] for generating medical scan pairs, and Cycle-Dehaze [55], a variant for dehazing images. Of important note, while the CycleGAN model can be used to convert images between a range of different domains, a specific model can only convert images between two domains at any given time. Conversion between additional domains would require another CycleGAN model to be trained for the additional domains. Alternatively, there are GAN models which are capable of multi-domain conversion, such as StarGAN [56], although performance naturally varies.

DALL-E 2 [57,58] and similar models, Craiyon (formally DALL-E mini) [59], Stable Diffusion [60], Parti [61], and Imagen [62], are all text-to-image generators. Text-to-image generators primarily consist of a network that can learn feature representations from text, typically a language model that can encode text into a latent space representation, and a generator model capable of mapping the text representation into an image, while GANs can be used in the generator side of text-to-image generators, other models have also been used to produce similar results. Text-to-image generators initially used VAEs to produce image outputs [63], but this later shifted to using GANs [64] and diffusion models for the same reasons GANs superseded VAEs for image generation. Most notably, Craiyon and Parti are the only models listed here that use GANs, with DALL-E 2, Stable Diffusion, and Imagen all use diffusion models in their image generation. Craiyon uses the CLIP network [65] for its text and image encoder and VQGAN [66] for its image generation. The primary reason for Craiyon’s use of VQGAN over other GAN models is its ability to synthesise high resolution images. GAN models have typically struggled to produce high quality images at higher resolutions due to the quadratic increase in cost in pixel space as the output resolution increases. VQGAN treats output images as a composition of smaller images, rich in perceptual information, allowing the generation of higher quality images at higher resolutions. The authors of the Craiyon model have noted that DALL-E, which is closed source, still provides higher quality images in comparison, but that Craiyon demonstrates the ability to generate reasonably good images at a much smaller resource scale. Parti uses ViT-VQGAN [67] in a sequence-to-sequence model, a common model in machine translation, where text tokens are transformed to image tokens. Similar to Craiyon, this allows Parti to synthesise higher quality, higher resolution images. Moving to DALL-E 2, Stable Diffusion, and Imagen, diffusion models function differently to GANs. Training is conducted by adding noise to input images in multiple stages, with the target being the reconstruction of the original image without noise. As more and more of the input image is replaced with noise, the diffusion model eventually learns to create images from nothing but noise. A language encoder is then used to generate noise maps from which images are synthesised, DALL-E 2 uses OpenAI’s GPT-3 model [68], Stable Diffusion uses the BERT-tokeniser [69], and Imagen has experimented with CLIP, T5 [70], and BERT. Diffusion models hold several advantages over GANs, the key ones being scalability and parallelisability, while avoiding the issues that come with adversarial training. These advantages may eventually lead to diffusion or hybrid models superseding GANs as the primary image synthesis model.

Deep conditional GANs (DCGAN), are a subclass of CNNs developed for unsupervised learning [71]. GANs as a model are known to be unstable during training, with mode collapse being a common failure point. In order to address this instability, various approaches have been taken. One such approach is DCGAN, which uses CNNs to stabilise the GAN model and learn unsupervised representations of image data. Unlike other GAN models, which typically have fully connected pooling layers, DCGAN replaces them with convolutional and transpose convolution layers that are not fully connected. DCGAN also uses batch layers, which are not present in other GAN models. A notable advantage of DCGANs is that its architecture makes it stable to train in most scenarios and less susceptible to mode collapse.

StyleGAN and its subsequent versions are a GAN model developed by researchers at Nvidia [72,73,74]. Initially developed as a combination of Progressive GAN [75] with style transfer, StyleGAN’s primary field is in the synthesis of human faces in resolutions up to 1024 × 1024 pixels. Starting with the base model, Progressive GAN learn by training the generator progressively from small resolutions, 4 × 4 pixels, up to 1024 × 1024 pixels, adding new convolutional layers for each increase in resolution. This method avoids the model instability that GANs typically run into when generating images at higher resolutions, while Progressive GAN itself is capable of generating a wide range of image classes, StyleGAN adapts it with style transfer for the specific goal of human face synthesis. Using GANs to synthesise human faces is a rather common application, but controlling the output of specific facial features has been a difficult task. Using Progressive GANs features, StyleGAN is able to build up features layer by layer, with each progressive layer controlling finer features without affecting coarser features built up in previous layers. To do this, the StyleGAN generator does not take points from a latent space as input, but rather, takes input from a noise layers and a standalone mapping network. The noise layers introduce stochastic variation in order to produce unique variations on features while the standalone mapping network controls which features are integrated in the different generator layers. StyleGAN divides layers into three feature grades, coarse features, middle features, and fine features. Coarse features, such as face pose and shape are generally defined by the lower resolution layers. Middle features, consisting of finer facial details and hair styles, are defined by the middle layers. Fine details, such as eye, hair, and skin colour are defined by the remaining layers up to 1024 × 1024 pixels. This division of features by layer allows specific features to be carried over between inputs when synthesising new faces. StyleGAN sees less use outside the generation of human faces, but within that field, it currently leads the generation of photorealistic human faces.

With the various and disadvantages presented by VAEs, GANs, and diffusion models, there have naturally been attempts to combine them to produce better image synthesis models. As with GAN models, there are a significant number of hybrid models. This review will briefly touch on two classes of hybrid models, VAE-GAN hybrids and Diffusion-GAN hybrids.

VAE-GAN hybrids [76,77,78,79,80] function by combining a VAE’s encoder into the GANs generator network, creating an unsupervised generative model capable of encoding, generating, and discriminating image samples. The goal of the combined network is to improve image sample quality, representation learning, sample diversity, and training stability compared to individual VAE and GAN models.

Diffusion-GAN hybrids [81] attempt to use noise diffusion, commonly used in diffusion models, to stabilise GAN models during training. By introducing a Gaussian mixture distribution to inject instance noise, the maximum noise to data ratio is adjusted over successive training steps resulting in a consistent performance over baseline GAN models in synthesising photorealistic images.

Overall, GANs have multiple notable use cases in synthetic data generation. The most common use case is to generate data for training other networks. This use case can be seen in a wide range of applications including face generation [74], medical applications [82], and sports camera calibration [83]. In the case of facial recognition, the performance of networks trained on synthetic data for facial recognition has historically not been very good with large domain gaps resulting in poor real world performance [31]. This issue can largely be traced back to poor intra-class variations causing lack of diversity and the domain gap between real and synthetic data. GANs can be used to blend faces together and vary domains to increase data diversity, improving training performance. Current medical research often suffers from biases in data due to the limited sample population that can be accessed. GANs offer the ability to synthesise some types of medical data such as skin lesions and cell images, forgoing the privacy concerns that usually come with using medical data [84]. The other use of GANs is as a sub network to a larger model, where models like CycleGAN can be used to adapt input data to the target domain during training, allowing both real and synthetic data to be adapted to the target domain without the input dataset needing to be modified directly [14,85].

Of course, GANs are not without their disadvantages either. Compared to other methods of image synthesis, GANs are comparatively weaker in generating good quality images at higher resolution. According to Nvidia [86] a GAN typically requires training data on the scale of 50,000 to 100,000 images in order to produce a high quality model, which can be difficult to obtain in some fields of research, or for smaller research groups. GANs are also not at the point where full 3D environment generation is possible, with 3D object generation being the furthest research into this particular area [87,88]. 2D video or animation generation is still in its early stages [89,90,91,92], meaning that GANs are not yet at the stage where data for computer vision tasks such as object tracking can be generated effectively. GANs are also a notably weaker when it comes to feature labelling within an image, while GANs are capable of producing specific class datasets, annotating specific features is a more complex task, while some specially trained GAN models are capable of object feature labelling, current text-to-image models may be able to generate an images of a large crowd, but it would not be able to provide annotations of the number of people present in the crowd and their locations within the image.

### 4.3. 3D Morphable Models and Parametric Models

3D morphable models (3DMM) are statistical models designed to generate facial shapes and appearances based on known relationships between the positions of facial features. 3DMMs were first introduced in 1999 [93] and have since seen significant development up to now, where it has been incorporated into state of the art face analysis models. 3DMMs are a generative model for faces built on the idea that all faces comprise of dense point-to-point correspondence and that face shape and colour should be disentangled from external factors such as lighting and cameras [94]. Because of the dense point-to-point correspondence, linear face combinations can be defined in order to produce morphologically realistic faces. The advantage of 3DMMs in creating synthetic faces over other models is that provides significantly more control over the generation of a face morph. Specific parameters can be adjusted to produce specific results and these parameters are not as hidden as what would be seen in a GAN model for face generation. Face models are also 3D, which means they can be used in a wider range of applications compared to 2D images.

In order to produce face morphs, 3DMMs need to have face capture data to train on. That said, while 3DMMs are used to generate 3D face morphs, 3D captures of faces are not a requirement and there have been attempts to learn 3DMMs from 2D datasets, although the difficulty does increase greatly when using data from the wild, which may not be captured under controlled conditions. Still, 3DMMs benefit from having 3D face capture data primarily because a 3DMM model needs at least three key pieces of information in order to produce faces, face shape, face appearance, and details of specific facial features, while not a core requirement for 3DMMs, dynamic face information is also starting to become a bigger part of 3DMM models as the field moves towards being able to animate traditionally static facial expressions. Current 3DMMs allow for significant modification of specific features, but the output usually leaves the face bland as a whole [95].

Face shape information is primarily obtained using one of three methods, geometric methods, photometric methods, and hybrid methods. Geometric methods capture face shape by either viewing it from two or more points and identifying corresponding points to build a face shape, or by identifying the correspondence between a projected pattern and an image of the projection. Photometric methods estimate surface orientation in order to reconstruct a 3D face shape. Hybrid methods use a mix of geometric and photometric methods to generate a face mesh. After collecting enough information to produce a face shape, an appearance needs to be layered on top of it, typical via textures. Theoretically, the mesh itself could hold appearance information, but in practice, it is easier to apply a texture map to the mesh. Textures can be as simple as colour projections from the source, but this method generally appearance properties to be tied to the illumination of the face when data was collected. Ideally, texture data should be captured from multiple cameras under diffused lighting conditions, but in practice this is difficult to achieve outside of very controlled environments. The final information required to produce a face morph consist of specific face features that do not translate well from typical texture maps, in particular, the eyes and hair. In the case of the eye, the cornea is transparent and distorts the appearance of the iris beneath it, which cannot be properly replicated using ordinary texture maps. Similarly, hair strands are not flat meshes and require specific approaches in order to create realistic looking results.

The difficulty in producing high quality 3DMMs has typically been in the collection of face data to create the model. Unfortunately, there are not a lot of large, publicly available, face datasets that contain the necessary information required to train a 3DMM. What datasets do exist tend to produce biased models due to the inherent bias in the base dataset. There has been research into countering dataset bias by using 3DMM generated faces to augmenting existing data sets to reduce bias [96], but this in itself requires a robust 3DMM model. Similar to StyleGAN, 3DMMs find their primary use in 3D face generation, but the model design itself does allow it to build parametric representations of many different objects on the basis that enough information is given to learn 3D object representations.

Parametric models are a more general method of data generation that refers to the use of parameterised variables to generate data from a theoretically finite number of possible instances. Compared to 3DMMs, parametric models see use in a wider range of fields. Parametric models can be challenging to create and are often limited by the data the models are built on. The model needs to ensure that the range of parameters provided is large enough to provide a diverse set of data without any parameters conflicting with each other to create unwanted results. In the case of face synthesis, with a large enough library of facial features and expressions, parametric models can offer the creation of a large range of human faces. There are also examples of parametric models being used in conjunction with 3DMMs to produce to create synthetic faces [15]. Semi-parametric image synthesis has been tested on datasets such as Cityscapes [97] and parametric models have also seen use in more obscure applications such as fish recognition [24], labelled photos of known fish were overlaid onto underwater background in varying positions, rotations, and sizes. Parametric model data generators allow for precise characteristics to be controlled, providing the ability to conduct quantitative studies.

### 4.4. Games, Game Engines and 3D Modelling Software

In recent years, the use of game engines and games to produce and collect synthetic data has become vastly more popular. Game engines such as Unreal Engine and Unity as well as 3D modelling software like Blender have seen more use in research as the tools and utilities they provide are vastly superior to creating a 3D virtual environment from basic code. The ability to create or use existing assets, capture scenes using virtual cameras, artificial lighting, and other functions virtual environments allowed virtual environments to be quickly set up and used [98]. Game engines have already been designed to handle tasks such as the development of virtual worlds and updates, additions, and plugins developed over time have made it even possible to directly link a game engine to a network for training [99]. Game engines can be used to generate large amounts of photorealistic data [9,11] for applications such as robotic simulation [8], satellite detection of planes [22], vehicle re-identification [32], and distracted pedestrian detection [100]. Full ground truth data can be obtained automatically automatically in most cases, which is very useful for applications such as stereo depth perception [101] and semantic segmentation [102] where manual annotation of data can be prohibitively time consuming.

When the functions provided by a game engine are not needed and a greater control over environmental models is required, 3D modelling software such as Blender provide alternative options. Compared to game engines, 3D modelling software provides better support for applications that might require manual modelling of objects [34] or interaction with other software that can directly interface with the modelling software as with MakeHuman and Blender [103]. Aside from 3D modelling, Blender is also capable of video editing and can achieve tasks such as motion tracking in both 2D video and 3D space.

In applications where specific data environments are not required, games can provide access to pre-built environments which can be used as is or modified to collect the necessary data. A prime example of collecting data from games are the datasets created from scenes within the game Grand Theft Auto V (GTA5) [13,14,104]. The datasets collected from GTA5 are predominantly crowd and pedestrian datasets as it is a comparatively easy method to gather free flowing pedestrian and crowd data without having to deal with privacy concerns, manually synthesising the required data, or building a system to automatically synthesise the data. This method of data generation also allows the collection of data on a scale that would be impractical or impossible in the real world within the same time frame.

Using games and games engines for synthetic data synthesis has several advantages over other data synthesis methods and even real data in some cases. A major advantage is that there is no need for training, not unless environment generation or asset behaviour needs to be automated in a way that does not use a parametric model. In the case of game engines, data can be collected as soon as the environment and assets are set up. Games forgo this entirely and data can be collected as simply as opening up the game and recording game play. These two methods of data synthesis also allow the tracking of ground truth data, which may not be available in other data synthesis methods and difficult to obtain in the real world. Object dimensions, classifications, movement, distances, and other details can all be collected from assets present in games and game engines. Not all that information may be necessary depending on the synthetic data requirements, but the benefit is that the information is available if it is needed. Video data can also be captured relatively easily in games and game engines in comparison to other data synthesis methods, where synthesising a video clip can be a complex and resource heavy task that may not even generate data to the required level of quality. There are also not many other options to generate 3D environments when it comes to image data synthesis. The primary limitation of building 3D environments in game engines is that a lot of the work still has to be done by a human, while some environments can be procedurally generated, that restricts the complexity of possible environments. Knowledge of game engine functionality and scripting is generally required to fully utilise a game engine. Games avoid this limitation at the cost of having less control over the environments as they are pre-built. Setting up a game to capture data may require knowledge of the game engine the game is built on as well as how to modify internal game files to set up specific scenarios which may not normally occur within the game. There is also the upfront cost of the game to consider as well as the end user agreements signed upon installing the game, which may restrict the usage and modification of game data, and therefore, restrict the applications in which data can be used, particularly if it is a commercial application.

### 4.5. Other Methodologies

There are data synthesis methods that do not fall into the previous categories, but their usage typically is not as widespread, either due to niche of data being synthesised or existence of more popular synthesis methods.

Line generation and rasterization algorithms used to overlay line data onto X-ray scans in order to produce training data for detecting catheters inserted into patients [105] are an example of an application niche that the synthesis method might not see wider use outside of this particular application.

Image based synthesis engines are rendering engines that take in large sets of data and extract known features from various images to produce a stitching of a new composite image that can look very unnatural to the human eye but contains the necessary feature information to train a computer vision algorithm. In the case of 3D human pose estimation [6], a set of real images with 2D annotations and a library of 3D motion capture data was provided to a image synthesis engine. By matching local joint poses in the 2D images to 3D motion capture pose data, the engine synthesised new images from parts of the 2D pose images that match the 3D pose, resulting in very unnatural looking images that were able to train models to similar performance levels as other methodologies. This can be considered an extension of overlaying 2D images where the images being overlaid are partial objects and environments instead of complete ones.

3D mesh reconstruction in CityEngine [28] was used to create datasets specifically with semantic segmentation and depth perception annotations. The resulting synthesised data looks highly unnatural from the ground perspective but has no significant display issues when viewed from above. This is an extension of texture projection onto meshes, but instead of the 3D environment being the goal, the goal is to reproduce the original dataset with additional segmentation and depth information that was not present in the original.

## 5. Performance of Synthetic Data

The performance of synthetic data in training is difficult to evaluate across multiple applications. Giving a definitive answer to the overall effectiveness of synthetic data in training from currently available findings is difficult if not impossible. However, comparing the performance of data synthesis methods within the same field is possible to a certain extent, given shared evaluation metrics, if not the same testing data sets. The following tables provide examples of the performance of synthetic data in different fields and comparisons to real data sets. There are two types of comparison, the first type are methods that generate synthetic data with the evaluation metric being how close a match it is to real data. The second type are dataset comparisons, looking at metrics one might consider when choosing a dataset to train, test, and validate a model. It is important to note that different papers use different synthetic datasets, validation datasets, training set sizes, validation set sizes, neural network models, and performance metrics, so performance across different fields should not be directly compared.

Table 1 shows the performance of different synthesis models for the task of synthesising faces as well as the output resolution and computational costs. This field of data synthesis is one of the few areas where relatively complete information on training time and costs is available from multiple papers. 3DMM models and parametric models have not been included in this comparison as there were not any models with a resolution, training time, or evaluation metric listed in the papers reviewed that could be used as a comparison. Interesting to note is the wide disparity between training time and synthesis time, with synthesis requiring significantly less time and computational power. At the same time, the computational cost can be seen as very large, in terms of time, power consumption, and hardware costs, with a Tesla V100 costing upwards of 10K USD on hardware release. That all said, the increase in time and computational requirements have resulted in significant improvements to synthesised data quality, both in terms of resolution and FID score. An important factor to note when comparing synthetic data performance as times goes on, the fid50k evaluation metric is already considered deprecated, with a newer version of the scoring method now used. However, it was the only evaluation metric uniform between the synthesis models listed, making it the best metric for comparison. In the future, older models may have to be rerun on newer evaluation metrics in order for any form of reasonable comparison to be made.

Table 2 compares the scale at which data can be synthesised compared to data collected from the real world. Fore the task of crowd counting, many real datasets are limited to low resolutions due to camera quality and dataset size due to the time required to capture data. In many real datasets, there is also very little control of how many people are present in a given frame. Publicly available real datasets with multiple scenes are also rarer, with datasets that contain multiple scenes having no consistent scenes between images. In comparison, synthetic crowd datasets are able to generate images at a much higher resolution and with greater control over scenes. The biggest advantages is the sheer number of images and people that can be captured from a synthesised environment. Capturing millions of people in a dataset for crowd counting would be an astronomical task if possible, let alone the manual annotation that would be required to label such a dataset.

Table 3 and Table 4 show the difficulty in comparing synthesised data in some fields, even where the application is the same. In the field of synthesising maps from aerial photos, the only common metric in evaluation was Turkers, humans hired to evaluate the images within a time frame and predict if the image was real or synthesised. Subjectiveness of the evaluation metric aside, the papers that listed the metrics did not list any additional information outside of the output resolution and performance. There was no information on training cost, training dataset, or anything else that could be used to compare and evaluate performance. Similarly, when comparing the performance of synthesising label maps from photos in the Cityscapes dataset, some papers do not provide any information outside of a given evaluation metric, making it very difficult to come to a reasonable conclusion regarding comparative performance. Overall, a great part of the struggle with synthetic data is the difficulty of evaluation, either due to limited overlapping metrics for comparison or lack of data to compare in general. It is possible to collect the source code of various models and run them on the same system to produce an even field for evaluation, but in practice, the amounts of data and computational data involved make such a task a massive undertaking that is not necessarily worth the resources used to do it.

## 6. Applications of Synthetic Data

In applications where real data is difficult to collect or annotate, the exact cause of difficulties may vary, applications may have legal issues with public data collection [84,103,105], require rare or specific events or environmental conditions to occur [2], are dangerous to collect in real life [100], be prohibitively difficult or expensive to collect [22], or be prohibitively difficult or expensive to annotate [1,13,32]. Synthetic image data can either serve to replace real data entirely as the training dataset or augment existing datasets to provide sufficient dataset size or offset existing data bias [11,96,120]. This section considers current usage in various fields and potential extensions of the use of synthetic data.

### 6.1. Object Recognition and Classification

Synthesis of image data for object recognition and classification comes in two primary forms. The first is static images where there is only one object or one type of object within the image. The objects themselves are not labelled within the image, but the image itself is given a classification for what it contains. The second form consists of images or videos that contain objects where the individual objects are labelled. The image or video itself are not classified, but the objects within it are, which gives a single image or video frame higher information density.

While public datasets for the latter are fewer, extensive public datasets for the former tasks already exist [20,121]. With large, publicly available datasets to train computer vision models, it may seem redundant to synthesise this type of data. In the case of classified images, particularly images synthesised by GANs, such data has seen use less as a replacement for real data but as a way of augmenting existing datasets, either to bolster dataset size [122,123] or to offset existing data bias [124,125]. With regards to performance and quality of synthesised data, existing datasets make it comparatively easier to direct comparisons in performance, allowing conclusions to be drawn regarding strengths, weaknesses, and potential improvements that could be made to data synthesis.

In the case of images with internal object labels, such data usually portrays more complex environments and has significant crossover with other computer vision applications, including urban driving [26,27], drone vision [28], and medical applications [84,103,122,123], which current public datasets may not fully cover the use cases of specific tasks. Synthesis of complex 3D environments is skewed towards the digital manipulation of real data [2] or capture of data from 3D environments [13,14] mainly due to due to the control those methods provide during the data synthesis process. That said, GAN does see use in synthesising medical scans due [122,123,126]. Most GANs less favoured for object labelled data compared to more parametric synthesis methods due to their lacking ability to label objects within the synthesised image as part of the data synthesis process. GANs and diffusion models have reached the point of being able to generate realistic scenes and objects based on word prompts, but even with the information of what the model is generating, the most GAN models are not designed label objects within images. GANs can be trained to detect objects [127], but use of such a model is still a separate network compared to the generator model. In comparison, parametric models or virtual worlds typically have all object information present in some way that can be used to annotate images post synthesis

With regards to existing datasets for object recognition and classification, despite their size, some research [128] has indicated that there are biases even in datasets like ImageNet with regards to how objects are displayed and presented which affects models trained on them. Using synthetic data to augment existing datasets can potentially improve data diversity and reduce any existing dataset bias [129]. Extension of GAN and diffusion model datasets to provide good quality internal image labelling, either by changing existing model architecture or by using a separate model in conjunction to label images [130]. Providing such information can greatly increase the range of applications that both GAN and diffusion models can be utilised in. Another logical extension of image synthesis for object recognition is object tracking, moving from images to videos [131,132]. Synthesising video data is more complex than synthesising images for some methodologies due to the need to maintain continuity from frame to frame while other methods already have such inbuilt capabilities. As a result, most video synthesis is done by capturing data from virtual worlds as opposed to generated from GAN or diffusion models. However, there are still opportunities to for those models to explore as the current time cost of generating data from 3D worlds is still high due to rendering requirements, particularly for photorealistic environments, whereas the cost for a GAN model to produce data is comparatively low once training is complete.

### 6.2. Face Recognition and Analysis

Face recognition and analysis is a field of computer vision that deals with the identification of people by their face from photos, videos, or real-time data streams. Its use falls primarily under biometric security and sees the majority of its use in security systems and law enforcement.

The benefits of using of synthetic data to train computer vision models for this application mostly comes down to the avoidance of legal issues that come with collecting face information from a large amount of real people. There are notable privacy concerns with the collection of photos or models of faces of real people [95] and, privacy aside, manually annotating human facial features is also a time consuming task [15]. Data collected from the wild can also vary greatly in quality [133], which in turn affects the quality of the trained model. Synthetic data can bypass most of these issues as any synthetically generated faces are, at most, similar to real people and are not directly copied from a real person. It is unavoidable that some synthesised faces will end up looking similar to real people, but as any similarities in generated faces are purely coincidental and there is no way to re-identify a human from a synthetic face. In the case of GANs, synthetic human faces can now be generated at a very large scale with each face being indistinguishable to from a real face to the human eye [74].

The training of a model to generate human faces ultimately still requires images or models of real humans to start with. In the case of GANs, the issue has mostly been resolved as GANs have advanced to the stage where they can generate realistic images of humans and feed the results back to itself or other models as training data. However, such GANs can currently only generate 2D images of human faces, at best, the same face can be viewed from different perspectives but not as a true 3D model [134]. For 3D models, 3DMM and parametric models still lead the field in face synthesis. However, in order to generate high quality models, 3DMM and parametric models typically have to use 3D face scans as a basis, which runs into the same privacy issue that made synthetic data appealing in the first place. 3DMM and parametric models also tend to generate faces that look stiffer and less realistic than what GAN models can produce. There is potential in using GANs to produce realistic images of a human face from different perspectives to feed into training 3DMM models, creating more realistic 3D human faces [135,136,137]. Beyond that, there is the potential to expand realistic face generation to human body generation [138,139,140], both 2D images and 3D models. Naturally, that also brings up concerns about the misuse of data, which will likely continue to become a bigger issue as image synthesis technology improves.

### 6.3. Medical Applications

Current use of computer vision in medical applications mostly applies to the processing of medical scans, but there is also work into vital sign detection from real-time streams [141,142]. Similar to facial recognition, there is a high level of difficulty in collecting large data sets for medical applications with regards to privacy. Even if it were possible to collect the required data without privacy issues, some types of medical data, such CT and MRI scans are prohibitively expensive to collect on a large scale [103]. Video data for applications such as vital sign detection runs into similar issues as sensors need to be attached to a person to record the vital sign information that needs to be paired with the video data. Legal and privacy issues aside, collecting such data is time consuming and manual labour intensive, not to mention having to align the recorded vital sign data with video data, as vital sign information is time sensitive.

Due to the sensitive nature of medical scans and personal health information, computer vision systems have yet to used in any major automated fashion with regards to tasks such as diagnosis. However, such systems still see use as support tools, which is where they benefit from synthetic data, allowing the training of models without using real data. For medical scans, GANs [122,123,126] and parametric models [105] have been main method of data synthesis. GANs are useful in generating general scan images while parametric models are more effective when specific features or objects within a medical scan have to be labelled. For vital sign data synthesis, this is a less explored field due to the application niche. Current data synthesis methods have primarily been on 2D images [143], but data collected from 3D environments and models may be the future for this area of application. Using 3D environments and models grants greater control over conditions like lighting and it is significantly easier to attempt to synthesise vital signs on a human model than to project what vital sign indications would look like onto a 2D model.

The medical field presents a great opportunity to explore the use of synthetic data. At the same time, the medical field also offers less margin of error compared to applications because of the nature of the data involved, meaning that the quality of synthetic data has to reach a certain level. For vital sign detection, the main issue is how to generate realistic looking images or models with synthetic vital signs, or at least to the level where the effect of domain gap can be reduced to negligible amounts via fine tuning. Most computer vision models require data to be under very controlled conditions as small changes in environmental conditions can greatly affect the accuracy of results. That said, synthetic data also offers the opportunity to train models to handle data from different environments without bearing the same risks as collecting a real dataset under such environmental conditions. Quality of synthesised data aside, there are concerns regarding the potential to fake medical documents, should synthetic data reach the level of being comparable to real data [84]. The limited availability of real data in some medical applications means that synthetic data stands to be a good alternative method of provide data for computer vision models and training data for humans. However, the problem of separating real data from fake data is something that will inevitably become more important as the technology develops.

### 6.4. Drone and Aerial Vision and Vehicle Automation

Computer vision for vehicles typically share one common factor, the processing of complex environments, either for navigation, object detection and identification, or both. With modern technology, it is not prohibitively difficult to collect data of road vehicles traversing through cities. There already exist high quality datasets which can be used to train computer vision systems for activities such as autonomous driving [27,49]. However, synthetic data is not without its uses in this application. As with most real data collection that potentially involves humans, there are privacy and safety laws that deal with who and what can be recorded [28].

Synthetic data provides a safe way to simulate such events and collect data that can be used to train models for such circumstances. One major example is to synthesis data for situations that would otherwise be dangerous in real life. Events such as objects, animals, or people suddenly cutting across a vehicle’s path or other vehicles disobeying road rules will happen in the real world [100]. However, it is not practically possible to capture such data in the real world and, even if it were possible, the danger it would pose to multiple parties makes real world collection of such data difficult. Another example is the use of drones in the field of search and rescue. Search and rescue operations are not scheduled events and, when a disaster occurs, collecting drone data to train a computer vision model is very low on the priority list, not to mention the ethical concerns of capturing footage of victims in disaster zones. Training for search and rescue operations do take place in areas designed to train personnel. However, such environments are usually limited in scope and, without a diverse set of data, it is difficult to train a computer vision model capable of supporting such work. Synthetic data offers the opportunity to provide the required type of data without the problems surrounding practical data collection and ethical concerns [144].

With regards to data captured from drones and planes, depending on the location, there are additional restrictions on where drones can be flown, how far a drone can be away from the pilot, and what altitude the drone can be flown up to. Drone flight is limited by the drone’s battery capacity as well as weather conditions, which makes synthetic data a promising alternative to collecting data from the real world. Aerial imagery captured from planes, helicopters or satellites face similar issues, with increased concerns in security in some cases. This is not to say that no drone vision or aerial imagery datasets exist [145,146,147,148], but the amount of publicly available data is significantly smaller compared to datasets available for other computer vision tasks such as object detection [20,121] or urban driving [27,49,149,150].

Complex environments such as cities are still possible to procedurally generate due, albeit with some restrictions on how the city might end up looking [151], but environments like disaster zones are significantly more complex and the nature of the complexity makes them difficult to generate. Further research to improve the generation of such environments could significantly speed up the synthesis of such data. The usefulness of realistic environments is also factor that has not been fully evaluated. Increasing the realism of a 3D environment also increases the computational cost of rendering and time to render. If highly realistic environments do not provide significant benefit in training, then realism could potentially be traded for faster data synthesis as well without compromising the training of a computer vision model.

### 6.5. Environmental Condition Synthesis

Among the many factors that can affect the quality of image data, environmental conditions are one factor in the real world that cannot be controlled. Conditions such as poor light, fog, and heavy rain might not be the best to collect data from. However, some applications require computer vision functionality in those situations, so it is necessary to have such data for those applications. However, it can be very difficult to obtain real data under specific environmental conditions such as fog [2], snow, rain, or different lighting conditions due to the lack of human control over them. Not only do the environmental conditions need to occur in the first place for it to be captured, equipment necessary to collect the data must also be present at the required location at the time.

Synthetic data allows data with specific environmental conditions to be generated, either by digital manipulation [53] or by modifying environmental parameters in a virtual world [152]. Video-to-video translation, an extension to image-to-image translation, is already possible for vehicle video data with regards to ambient lighting [153] and CycleGAN is capable of translating images between environmental domains. Extension into environmental effects allows for the creation of datasets with much larger environmental diversity than might have been possible in the real world as well as data that would be riskier to collect in the real world, such as heavy storms or fog.

There is potential in not only augmenting existing datasets or adapting existing datasets into new datasets, but to integrate into other fields of computer vision, such as drone vision. This area of data synthesis has primarily found use in navigation applications, but such data could also see use in creating paired datasets to train computer vision models to remove environmental conditions, providing an estimation of scenes without conditions without obscuring environment effects.

### 6.6. Human Detection, Crowd Counting and Action Recognition

Human detection, crowd counting, and action recognition can all be considered adjacent fields of application for computer vision. Human detection and crowd counting tasks are primarily concerned with identifying location and total number of people within an image or video while action recognition deals with specific actions that a human might be carrying out, while the collection of data for human detection does have to deal with both privacy concerns and manual annotation of data [5,104], those difficulties aside aside, collecting real human and crowd data is not that difficult. The main difficulty comes from the manual annotation of data, which can be very tedious, leading to increases in human error as the dataset set increases and degradation in dataset quality. Both problems have resulted in a lack of large annotated data sets for training computer vision models. Synthetic data can be used to bypass both problems and can also be used to generate data sets at a larger scale than what might have been able to be collected from real locations.

Crowd counting is a good example of data sets that are difficult for humans to annotate, while time consuming, small crowds with clear figures may still be possible to accurately annotate by hand. However, when crowd densities reach high levels and figures start to become highly obscured, time requirements and rates of human error increase significantly. This is particularly true when pixel-wise positions of humans are required and not just bounding boxes [13]. Synthetic data is useful in this application as the exact positioning of human figures can be recorded as they are placed into a synthetic crowd [115]. In the case of virtual worlds or data captured from games, the 3D position of the human can also be recorded, providing significantly more information that can be used to train a model [14,117].

Human action recognition is effectively an extension of human pose recognition across multiple image frames, where pose recognition is identification and classification of joints in the human body. A given sequence of human poses can subsequently be used to identify what kind of action a person is doing or if they are doing any action at all, while there are many publicly available datasets that contain humans, few are annotated with the labels required for human pose and action recognition [6,21]. This is particularly an issue with 3D pose estimation, where even if it is possible to annotate existing data sets with pose estimations manually, in most cases only 2D poses can be augmented. Synthesis of human action data has the benefit of knowing where joints used in pose recognition are as a 3D human model needs to have a skeleton rigged in order to conduct any motions at all, allowing for pose guesses to be cross checked against a reliable source.

While this field is primarily concerned with humans, as the name indicates, the methodologies used have potential to be extended to objects other than humans, whether they be vehicles, machines, or animals.

### 6.7. Depth Perception and Semantic Segmentation Tasks

Depth perception and semantic segmentation are both computer vision tasks that require data with significant annotation to be useful. However, such data is expensive to obtain in large quantities [10] and manually annotating such data is laborious and time consuming [1,154]. In the case of stereo imagery, humans have difficulty classifying such data accurately [155]. This has resulted in an overall lack of large publicly available datasets for these applications.

Synthetic data provides two key benefits for both tasks. Firstly, synthetic data can provide locations and distances of different objects with pin point accuracy. Secondly, data can be generated on a much larger scale. This covers the two major issues facing the field with regards to the lack of large data sets and the cost of manual annotation, while this is an extensive field in its own right, synthesis of such data can potentially be extended to the integration of such information into datasets in other applications that currently do not have such information.

## 7. Research Gaps and Challenges

Despite the promising results of synthetic data up until now, there are still many research gaps and challenges. Past surveys [16,19,156,157] have noted several keys areas of discussion, including synthetic data domain gaps, data diversity and bias, and synthetic data benchmarking. In addition to theses areas of discussion, the additional points of the effectiveness of photorealistic data and the computational cost of creating synthetic data is also considered.

### 7.1. Domain Gap

Domain gap refers to the gap in relationship between data sets collected from different domains. The domain gap between real and synthetic data has been acknowledged in many research papers and significant work has been conducted into how the gap can be minimised as much as possible. Some domain gaps can be identified relatively easily, such as the difference between datasets collected in bright lighting conditions and dark lighting conditions. However, while present and significant in some cases, the domain gap between real and synthetic data can often be harder to identify. The domain gap between real and synthetic data often results in large gaps in performance on models trained purely on synthetic data. Tsirikoglou, et al. [16] noted that no models trained purely on synthetic data did not perform better than models trained on real data when tested on real data, with the process of data synthesis itself likely affecting the domain gap. In many cases, fine tuning on real data significantly improves the performance of the model. However, this does not solve the fundamental issue of the domain gap. The two most common approaches to dealing with the domain gap between real and synthetic data have been to either mix real data into the training set alongside synthetic data and fine tuning on real data after pre-training on synthetic data or to improve the synthetic data itself, moving its domain closer to the target domain. With current methodologies, fine tuning with real data is significantly simpler, but improvements to synthetic data quality will likely be the solution in the long run. With regards to improving synthetic data, they have been primarily focused on either increasing the photorealism of the data or improving synthetic data diversity in order to provide better domain generalisation.

### 7.2. Data Diversity

Somewhat tied to domain gap, data diversity deal with the quality of data and how well models trained on a given dataset generalise to other domains. Lack of data diversity is a known issue with synthetic data generation as some methodologies have been found to easily produce biased data sets under some input conditions. Models trained on diverse data sets typically show higher performance when processing data outside the target domain. At the same time, if a data set is diverse but there are not enough instances of specific domains, the resultant model could potentially fail to deliver good results in any domain. Data diversity is also something that is difficult to measure and is also less tangible compared to photorealism. Tsirikoglou, et al. [16] considers that real data will inevitably skew synthesis data due to model training, with the only way to avoid it being to not use real data for training at all. However, large scale data synthesis does offer the opportunity to create training samples which reduce the inherent data bias. As a whole, evaluating data diversity has become easier [19] with the introduction of various data scoring methods such as IS [45] and FID [106]. However, most such methods are more focused on the evaluation of the synthesis model itself and less on the synthesised images. Future evaluation methods on synthesised data may have to consider data quality of individual images in order to build up a dataset diversity score as opposed to evaluating the dataset as a whole. Data diversity is an important factor when considering the quality of datasets in general, whether real or synthetic, and improvements to evaluating data diversity are always welcomed, more so if the method is applicable in multiple fields.

### 7.3. Photorealism

In data synthethsis, photorealism is goal of making synthesised data look as close to what would be captured in the real world, the core idea being that training on photorealistic data will reduce the domain gap to the target domain. This applies not only to people, but also to objects, environments, and environmental effects. Photorealism has been pointed to as a potential method of improving synthetic data quality [16], but the exact benefits are still being debated with different papers showing varying results of the impact of photorealism on the performance of synthetic data [8,30]. Some fields are also known to benefit from photorealism more than others with face detection and analysis being one of them. The impact on other applications is less clear with some research indicating that domain randomisation of data, with less realistic environments, could also improve data quality [30]. Naturally, there is also a trade off with photorealims, with increases in photorealism typically coming at the cost of higher computational requirements. This is a subject that has not seen much discussion, partially due to the lack of information regarding computational resource requirements, but whether increases in photorealism and computational resource requirements are worth the improvement in performance is something that will have to be considered in future work.

### 7.4. Computational Resource Requirements

Computational resource requirement is a major factor in synthetic data generation, but few papers seem to provide information on the computational resources and time used to generate a given synthetic dataset. The StyleGAN papers have been the most consistent, with information on hardware used and time to train recorded as party of the research done [72,73,74]. On the other hand, other papers on data synthesis usually have no mention of hardware and training time [14,109,117,118]. Often, there may only be a comment on the hardware used to generate the data and whether the hardware was provided as part of sponsorship or donation [48,50,107]. However, this information is important when it comes to evaluating methodologies for generating synthetic data. For larger research groups or businesses, this may not be a major concern as they have the resources to synthesis data within reasonable time frames. The StyleGAN-3-T model took over a week to train, utilising 8 high end Nvidia GPUs [74] that would have costed upwards of 50k USD to purchase, ignoring the rest of the system needed to support such GPUs. Using only one of the GPUs listed in the StyleGAN paper would have required a training time of over 2 months. The lack of this information means that it is difficult to gauge the practicality of some data synthesis methods as the computation and time requirements can greatly restrict, restricting the amount of data that can be practically generated. Performance of the synthetic data being equal, data generation efficiency becomes the next important factor. Methods that utilise large server farms to generate data sets may not be practical for applications that do not have readily available access to such resources. A data set that takes days on a server farm may take weeks or months on lower computational power hardware. This information can be used to weigh the benefits of generating synthetic data over collecting real data. In some applications, the cost of generating high quality synthetic data might be greater than the cost of manually collecting and annotating real data, although this is hard to judge due to the lack of existing data on the matter. Moving forward, there is not a significant amount of information that needs to be included to be useful, a brief mention of the hardware used and the time taken to generate a given number of data instances is enough for basic comparisons and evaluations to be made.

### 7.5. Synthetic Data Benchmark

Lastly, the effectiveness of synthetic data has been difficult to gauge primarily due to the lack of a central method of benchmarking synthetic data. Current dataset benchmarks exist to benchmark computer vision models and not the data itself, with training done on synthetic data and evaluation done on real data. This method of benchmarking makes sense as the aim of any computer vision model is to be used on real data [16]. However, this has resulted in lacking methods to benchmark datasets in general, let alone synthetic datasets. Many different methods of benchmarking data exist, ranging from detection based scores, classification accuracy, precision and recall metrics, diversity scores and many others [19]. However, all those methods of scoring data are not necessarily cross compatible between datasets, making it difficult to make comparisons between datasets. Xue, et al. [156] suggests that a standard process could be established for different areas of data synthesis to allow for fair benchmark comparisons, but creating a universally agreed upon benchmark will likely still be difficult, if the existing number of metrics is anything to go by. Luo [157] notes that selecting a good evaluation metric for a given data synthesis model is still a challenging task and that better methods of evaluating image quality are needed in general.

Of the papers on synthetic data that were reviewed, Virtual KITTI [29] was the only notable example of synthetic replica of a real data set, allowing a direct comparison to be made. The results of the evaluation of the synthetic copy indicated that for the purposes of multi-object tracking from the perspective of a road vehicle, training on synthetic data and fine tuning on real data produces similar results to training on real data. However, these results have no bearing on the effectiveness of synthetic data beyond the application posed in the paper. There is also no indication of how effective a synthetic dataset in the same style as Virtual KITTI but of different scenes that were not present in the real data might have on performance.

Until now, computer vision has been focused around the improvement of the neural network model performance, when provided with the same training data set. The effectiveness of synthetic data is instead concerned with the improvement of data quality and comparing the data quality to real data, with the goal of improving model performance via improvements in data quality, assuming the model remains the same. Because of this, the evaluation of the effectiveness of synthetic data needs to be carried out with the idea that the algorithms used to gauge performance are the constant and that iterations to the synthetic data generation methodology are what needs to change.

A possible solution to this issue would be to be to select several state of the art computer vision algorithms to serve as a model benchmark along side matching real datasets. Data quality would then be based on the performance of the model trained on a given dataset compared to the benchmark dataset. Naturally, this method is susceptible to model and dataset bias depending on the model and dataset chosen to serve as a benchmark, but it still offers an equal foundation from which datasets, both real and synthetic, can be evaluated.

## 8. Conclusions

As computer vision develops further and requirements on data increase, synthetic data stands as a potential alternative to real data in many applications where collection of real data is expensive. The current performance of computer vision models trained on synthetic data might not match real data in any applications, but its performance in some fields shows that synthetic data has the ability to at least augment existing data sets if not replace them. There is still a notable domain gap between synthetic data and real data in most applications and the effect of photorealism and data diversity on the performance of synthetic data are not fully known, but clear directions exist in which progress can be made towards evaluation. As research into data synthesis grows, the need for complete information on synthesis models becomes more and more important, with regards to training data, training costs, and evaluation metrics, information that is currently not provided in many papers. Creating a framework for the evaluation of not just synthetic data, but data in general, will likely become an important step in the progress of data synthesis.

## Figures and Tables

**Table 1:** Performance of different data synthesis methods for face synthesis, the lower the FID score the better.

Method	Output Resolution	Training Cost	Generation Cost (50k Images)	FID Score [106] (fid50k)
Pix2Pix [50]	256 × 256	N/A @ 1xTitan X	N/A @ 1xTitan X	112.01
Pix2PixHD [107]	256 × 256	N/A @ 1xTitan X	N/A @ 1xTitan X	23.89
CycleGAN [48]	256 × 256	N/A @ 1xTitan X	N/A @ 1xTitan X	19.35
Graph2Pix [108]	256 × 256	N/A @ 1xTitan X	N/A @ 1xTitan X	19.29
GNARF [109]	256 × 256	N/A	N/A	7.9
EG3D [110]	512 × 512	8d 12h @ 8xTesla V100	N/A @ RTX 3090	4.7
StyleGAN-1 [72]	1024 × 1024	6d 14h @ 8xTesla V100	16m @ 1xTesla V100	4.41
StyleGAN-2 [73]	1024 × 1024	9d 18h @ 8xTesla V100	22m @ 1xTesla V100	2.84
StyleGAN-3-T [74]	1024 × 1024	8d 5h @ 8xTesla V100	Up to 1h @ 1xTesla V100	2.79
StyleGAN-3-R [74]	1024 × 1024	1d 17h @ 8xTesla V100	Up to 1h @ 1xTesla V100	3.07

**Table 2:** Comparison of Real and Synthetic Datasets for Crowd Counting.

Method	Output Resolution	Dataset Size	Scene Locations	Pedestrian Instances
UCSD [111] (Real)	238 × 158	2000 images	1	49,885
Mall [112] (Real)	640 × 480	2000 images	1	>60,000
PETS2009 [113] (Real)	576 × 768	875 images	1	4307
ShanghaiTech [114] (Real)	576 × 768	1198 images	N/A	330,000
CrowdFlow [115] (Synthetic)	1280 × 720	3200 images	5	<1451
CrowdHuman [116] (Real)	<1400 × 800	24,370 images	N/A	470,000
GCC [14] (Synthetic)	1920 × 1080	15,212 images	100	7,625,843
GCC [117] (Synthetic)	1920 × 1080	280,000 images	31	∼38,000,000

**Table 3:** Performance of different data synthesis methods for aerial maps, photo to map, the higher percent of Turkers that classify the output as real, the better.

Method	Resolution	Training Cost	Generation Cost	% Turkers Labeled Real
CoGAN [118]	512 × 512	N/A	N/A	0.6 ± 0.5%
BiGAN/ALI [77,119]	512 × 512	N/A	N/A	2.1 ± 1%
CycleGAN [48]	512 × 512	N/A	N/A	26.8 ± 2.8%

**Table 4:** Performance of different data synthesis methods for image segmentation against the Cityscapes photos to labels dataset, the higher the per-pixel accuracy, the better.

Method	Resolution	Training Cost	Generation Cost	Per-Pixel Accuracy
BiGAN/ALI [77,119]	N/A	N/A	N/A	0.41
CoGAN [118]	N/A	N/A	N/A	0.45
CycleGAN [48]	N/A	N/A	N/A	0.58
Pix2Pix [50]	256 × 256	N/A	N/A	0.85
Pix2PixHD [107]	2048 × 1024	N/A	N/A	0.83

## Abbreviations

The following abbreviations are used in this manuscript:

CNN	Convolutional neural network
3DMM	3D morphable models
FID	Frechet Inception Distance
GAN	Generative adversarial network
GPU	Graphics Processing Unit
GTA5	Grand Theft Auto V
IS	Inception Score
VAE	Variational Autoencoder
